# Transanal endoscopic micro-surgery (TEMS) for the management of large or sessile rectal adenomas: a review of the technique and indications

**DOI:** 10.1186/1477-7800-3-13

**Published:** 2006-05-04

**Authors:** Savvas Papagrigoriadis

**Affiliations:** 1 Department of Colorectal Surgery, King's College Hospital, London, UK

## Abstract

In this review article the surgical technique of Transanal Endoscopic Microsurgery (TEMS) is examined. A number of techniques have been used to treat adenomas of the rectum. The treatment of large adenomas which occupy a large surface of the rectal lumen or adenomas which are flat and grow in a "carpet-like" fashion is particularly challenging. Major rectal surgery carries a risk of morbidity and mortality, particularly in elderly and unfit patients. Although local excision with transanal resection (TAR) and the Kraske sacral operation have been used in the past, during the last twenty years TEMS has become the method of choice for those lesions. TEMS is efficient and minimally invasive. The technique allows the patient to recover rapidly and the incidence of complications is much lower than that of major surgery. In case of recurrence the option of repeat TEMS or major surgery remain available. TEMS has been slow to gain popularity mainly for reasons of cost and steep learning curve but it is now an established procedure and a valuable therapeutic option which is particularly useful for elderly and unfit patients. Gastroenterologists should be aware of the nature and indications of TEMS in order to advise and refer selected patients with rectal adenomas accordingly.

## Rectal adenomas and the dilemmas of surgical treatment

Adenomas of the rectum are a common condition since most rectal cancers are the end result of the adenoma – carcinoma sequence. All rectal adenomas should be excised or ablated since it is well established that gradual increase in the size of the adenoma carries a parallel risk of malignant mutation and development of cancer. Apart from the risk of malignant transformation these lesions require treatment for relief of symptoms. They can cause rectal bleeding, tenesmus and discharge of mucous which can be so profound that it can lead to dehydration and hypokalaemia.

Small adenomatous polyps are successfully treated by snaring or destroyed by "hot" diathermy biopsy. Larger adenomas are still amenable to snaring if they have a stalk around which a snare can pass.

The problem arises with two categories of adenomatous polyps. The first one is the villous adenomas, a well differentiated variety with lower malignant potential which tends to grow to a large size, often occupying most of the circumference of the rectum or extending along the lumen over a distance of several centimetres, creating a long tumour. Very large size prevents snaring not only for technical reasons but also because of high risk of severe bleeding at the end of the procedure.

The second category is adenomatous lesions, which although they are not particularly large, have a flat base and grow in a "carpet-like" fashion. Those lesions cannot be snared and alternative ablation methods, such as Argon beamer coagulation, have been described. Ablation methods work temporarily and have a low complications rate; however there is a high incidence of recurrence[1] and the average patient needs a large number of follow-up endoscopies and repeat treatments. Occasional lesions are too extensive to submit to ablation treatment.

The last resort for those two categories of difficult rectal benign lesions has been the same treatment that is applied for rectal cancer: radical surgery. The disadvantage of radical rectal surgery is that it consists of complex major operations which carry a significant risk of mortality. The mortality risk is typically quoted as 2–5% but this is an average figure which is higher for certain groups of patients. Rectal adenomas often occur on the elderly and there is an exponential increase in peri-operative risk with age. Patients over the age of 80 who undergo anterior resection of the rectum have a 15% risk of peri-operative death because they either respond poorly to treatment of common surgical complications such as chest infections etc. or are subject to a high risk of developing cardiac events, cerebro-vascular events etc.

Radical surgery of the rectum is also risky for patients who have comorbidities. The risk in association with the American Society of Anesthesiology Grading has been studied extensively. While peri-operative risk of death is 0.5% for patients who are ASA Grade I (healthy individuals) this risk jumps to more than 25% for patients ASA Grade IV (patients with comorbidities which are not adequately controlled) [2-4]. It is for the latter patients that major rectal surgery is a risky option and a less invasive treatment is required.

The other important consideration is the impact of radical rectal surgery on the patient's quality of life. Elderly people's health status often functions on a fine balance which can be upset by the assault of major surgery. Post-operative delirium and aggravation of pre-existing dementia has been increasingly recognised as a side effect of major surgery in the elderly [5].

Major rectal surgery has sometimes an impact on quality of life because of side effects of bladder dysfunction or erectile dysfunction [6,7]. Both those complications can affect adversely the quality of life and patients have to be warned regarding the risk.

Rectal surgery carries the risk of a stoma: a permanent colostomy in the case of an abdominoperineal resection of the rectum or a temporary ileostomy or colostomy in the case of an anterior resection of the rectum. A temporary stoma carries the necessity for one more operation to close it. The presence of a stoma can be both hard to accept and difficult to manage for some patients.

There are also possible social implications: more than one quarter of people over 70 in the UK live alone and the elderly often take as long as 2–3 months to recover after major abdominal surgery, particularly if complications have occurred.

## Surgical procedures

From the above it is clear that although major rectal resection is the most effective way of curing a large or sessile rectal adenoma it is a method which also has considerable disadvantages. Less invasive local excision methods have been devised in order to minimise morbidity and mortality.

Three methods have been described:

• Trans-anal resection (TAR),

• the Kraske perineal or sacral resection

• Trans-anal Endoscopic Micro-Surgery (TEMS).

### Transanal resection

Transanal resection has been practiced for a long time [8]; but is successful for lesions which have both their lower and upper margins lying within 5 centimetres from the anal verge. Access to the lesion is by dilating the anus by means of anal retractors. Infiltration of the base of the adenoma with adrenaline allows mucosal excision with minimal blood loss. For low lesions Trans-Anal Resection (TAR) is a satisfactory technique and good results have been described in the literature. The problem is that it is a suitable technique only for a small number of adenomas which lie entirely in the lower rectum.

### The Kraske perineal or sacral rectal resection

This is an old technique which is not currently practiced widely [9,10]. It involves a trans-coccygeal approach of the rectum posteriorly, opening of the rectum, excision of the tumour and re-suturing of the posterior aspect of the rectum. It can have a high incidence of complications such as perineal abscess from suture line leak and this may explain why it has not become popular.

#### Transanal Endoscopic Microsurgery (TEMS)

This procedure was first described and developed by G. Buess in the early eighties [11,12]. It requires specially designed equipment which until recently had a high cost. TEMS also requires a surgeon who possesses advanced laparoscopic skills since it is essentially a form of laparoscopic surgery performed in a much more confined space (inside a cylinder of 4 centimetres diameter as opposed to the abdominal cavity). The technique is therefore demanding and one of the problems is that the learning curve is steep because the number of cases is (or has been so far) rather small for surgeons to acquire technical expertise. Concentration of cases in certain centres would allow for easier accumulation of experience with the technique.

## TEMS: the procedure

The equipment necessary for TEMS is shown in Picture 1. This consists of the operating 4 centimetres diameter sigmoidoscope, the 0 degree telescope, laparoscopic atraumatic forceps, laparoscopic diathermy or vessel sealer, laparoscopic irrigation-suction device. The above instruments are connected to a standard laparoscopic "stack" incorporating a gas source, a light source and a high resolution monitor.

Full bowel preparation is required pre-operatively. The patient is put in lithotomy position and the whole procedure is performed transanally unless there is a (rare) complication of intra-abdominal perforation of the rectum. General anaesthetic is used mostly although the author and others have performed cases under spinal anaesthesia. The duration of the procedure depends on a number of technical factors such as size and height of the lesion as well as factors to do with the equipment and can vary from 30 minutes to 3 hours. After the operation the patient can drink and eat immediately and can receive oral analgesia without the need for pareneteral opiates. In most cases discharge is within 24 hours. Temporary minor urgency incontinence may occasionally occur for a few days, and although laboratory measurements of anorectal function are altered short term, in all so far reported series there is no problem of long term incontinence [13-18].

There is a small but material risk of intra-operative complications which usually have to do with technical aspects of the procedure so careful selection of patients and attention to technical details is mandatory. The height of the tumour from the anal verge is important: TEMS is safe only in cases of tumours which lie extra-peritoneally. If resection of tumours of the distal sigmoid is attempted there is a high risk of intra-abdominal perforation and need for laparotomy. Bleeding is common and can sometimes be difficult to control. The introduction of equipment such as the ultrasonic scalpel and the Ligasure diathermy vessel sealer seems to be a significant advancement in prevention of severe bleeding.

In all series the incidence of complications during TEMS is lower than that of major rectal surgery [19-25]. More importantly there does not seem to be yet any reported mortality from TEMS, although this may change with the expansion of the indications to include more elderly and unfit patients.

Recurrence of rectal adenomas after TEMS is not frequent but still possible as several series have indicated [26]. Repeat TEMS in those cases is possible [27]. When excision of adenoma with TEMS reveals the presence of cancer in the specimen there are two options: either "salvage" major surgery or additional treatment with radiotherapy and/or chemotherapy [28,29]. Although the examination of the role of TEMS in the treatment of rectal cancer is beyond of the scope of this article, impressively good results have been occasionally described in treatment of cases of early carcinomas with additional radiotherapy [30,31]. This indicates that suspicion of the presence of carcinoma within a large rectal adenoma is not a contraindication for application of TEMS as first line treatment until finalisation of the histology.

## Conclusion

TEMS is a useful minimally invasive technique for treatment of certain large or sessile adenomas of the rectum. It can successfully treat those adenomas which are not amenable to colonoscopic excision and can spare some patients the risks and side effects of major rectal surgery. It can be preformed as a short stay procedure even without general anaesthetic, with minimal morbidity and no mortality. In case of malignant transformation or recurrence it is still worth doing TEMS as first line treatment since it does not preclude radical surgery and can be repeated for treatment of recurrences. Gastroenterologists should be aware of the usefulness of TEMS as a therapeutic option in order to advise and refer their patients accordingly.
